# Selection and characterization of a human ovarian cancer cell line resistant to auranofin

**DOI:** 10.18632/oncotarget.21708

**Published:** 2017-10-09

**Authors:** Ida Landini, Andrea Lapucci, Alessandro Pratesi, Lara Massai, Cristina Napoli, Gabriele Perrone, Pamela Pinzani, Luigi Messori, Enrico Mini, Stefania Nobili

**Affiliations:** ^1^ Department of Experimental and Clinical Medicine, University of Florence, Firenze, Italy; ^2^ Department of Chemistry “Ugo Schiff”, University of Florence, Firenze, Italy; ^3^ Department of Health Sciences, University of Florence, Firenze, Italy; ^4^ Department of Experimental and Clinical Biomedical Sciences, University of Florence, Firenze, Italy

**Keywords:** auranofin, tumour drug resistance, human tumour cell lines, drug effects, gene expression

## Abstract

The anti-arthritic drug auranofin exerts also potent antitumour activity in *in vitro* and *in vivo* models, whose mechanisms are not yet well defined. From an auranofin-sensitive human ovarian cancer cell line A2780, a highly resistant (>20-fold) subline (A2780/AF-R) was developed and characterized. Marked reduction of gold accumulation occurred in auranofin-resistant A2780 cells. Also, moderately higher thioredoxin reductase activity in A2780/AF-R cells was observed while no changes in intracellular glutathione content occurred. Resistance to auranofin was associated with a low level of cross-resistance to some investigational gold compounds as well as to oxaliplatin and other anticancer drugs with different mode of action (i.e. melphalan, vinblastine, doxorubicin, etoposide, and paclitaxel). Reduced gold accumulation was associated to substantial gene expression changes in various influx (e.g. *SLC22A1, SLC47A1, SLCO1B1*) and efflux (e.g. *ABCB1, ABCC2, ABCC3*) transporters. The expression levels of selected proteins (i.e. SLC22A1, SLC47A1, P-gp) were also changed accordingly. These data provide evidence that multiple drug transporters may act as mediators of transport of auranofin and other gold compounds in cancer cells. Further investigation into the molecular mechanisms mediating transport of auranofin and new gold complexes in view of their potential clinical application in the treatment of cancer is warranted.

## INTRODUCTION

Auranofin, an oral gold (I) compound, was developed in the 1980's for the treatment of rheumatoid arthritis [1]. Despite its appreciable efficacy in the treatment of this disease, auranofin is at present rarely used in the clinic since novel and more effective anti-rheumatic drugs have become available [2, 3]. Yet, auranofin in recent years has shown promise in the treatment of several different diseases, including cancer. Preclinical evidence in various tumour models [4–7] have contributed to the implementation of clinical trials of auranofin in different types of tumours (e.g. leukaemia, ovarian cancer and other solid tumours) [8]. Most of these studies are based on programs of drug repurposing promoted by the National Institute of Health (NIH) [9] and other institutions [10, 11].

It is now ascertained that auranofin exerts potent antineoplastic activity in various *in vitro* and *in vivo* tumour models [12–14]. It also represents a reference compound when new gold-based compounds are studied as potential anticancer drugs.

The mechanism of action of auranofin in cancer is not fully understood. Auranofin was shown to cause a dose-dependent inhibition on DNA, RNA and protein synthesis in human tumour Hela cells and murine B16 tumour cells at cytotoxic concentrations [4, 5]. However, subsequent studies investigating the role of DNA replicative enzymes failed to show that the inhibition of DNA replication is a critical factor in the cytotoxicity of this agent [15]. Thus, it was recognized that auranofin may interact with several other cellular macromolecules leading to injures to diverse cell structures and inhibition of a number of essential signalling pathways involved in the progression of cancer [12, 16]. However, unlike cisplatin, extensive evidence showed that cytotoxicity of auranofin is not due to a direct interaction with DNA [17].

The antineoplastic activity of auranofin relies on inhibition of cell proliferation by induction of apoptotic cell death and block of tumour neoangiogenesis [12, 13]. Auranofin was shown to induce apoptosis in several different cancer cell lines through increased production of reactive oxygen species and by modification of cell redox status [18, 19]. In addition, as other gold compounds, auranofin inhibits mitochondrial [20] and cytosolic [21] thioredoxin reductase leading to oxidative stress including peroxiredoxin 3 oxidation [18], and consequent cellular accumulation of H_2_O_2_ which triggers Bax-Bak-dependent apoptosis [18, 22]. A further consequence of thioredoxin reductase inhibition is the disruption of selenoprotein synthesis that leads to the disruption of DNA synthesis [23].

Also, auranofin inhibits several tumour signalling pathways, independent pro-inflammatory and neoangiogenesis pathways, contributing to the control of cancer growth and progression [13]. As several other gold compounds, it has been suggested that auranofin targets the proteasome [12]. Notably, in tumour cells, auranofin does not inhibit the three main catalytic peptidase activities of purified 20S proteasome [24] but inhibits 19S proteasome-associated deubiquitinases, consequently inducing cell apoptosis [25, 26]. Overall, these observations suggest for auranofin a complex and multifactorial mode of action.

To further improve the understanding of the molecular mechanisms of auranofin cytotoxicity as well as to unravel the mechanisms responsible for resistance to gold compounds, the development and characterization of auranofin-resistant cell lines may be useful. Very few studies have so far addressed these issues [27, 28] and none of them were accomplished in human tumour cell lines. Indeed, experimental studies using relevant *in vitro* and *in vivo* models of drug-resistance and drug-sensitive human cancer may lead to improved knowledge of the mechanisms of action and resistance of gold compounds as well as of the strategies to develop gold-based analogues of potential clinical utility.

We describe here the development and characterization of an auranofin-resistant human ovarian cancer cell line (A2780/AF-R) which was established from an auranofin-sensitive parental cell line (A2780). Properties of this resistant line were compared with those of the parental line. Resistance to auranofin and cross-resistance to several other drugs including recently synthesized gold (I) and (III) compounds, were determined. Furthermore, in these cell lines, we examined differences in cellular gold content and in the expression of proteins potentially involved in cell influx/efflux of auranofin as well as the potential reversion of auranofin resistance by verapamil.

## RESULTS

### Characteristics of the auranofin resistant cell line

Parental A2780 cells were cultured in the continuous presence of stepwise increasing auranofin concentrations. Following a six-step rise of auranofin concentrations from 0.1 to 7.0 μM in 8 months, a resistant subline was obtained (A2780/AF-R). At this stage, a resistance index (RI) of about 22-fold was obtained and the selected cells were able to grow at 7 μM auranofin, a concentration at which no cells of the parental line survive. In monolayer culture the A2780/AF-R cell line had a doubling time similar to that of the parental A2780 cell line (22.60 ± 2.58 and 19.70 ± 4.66 hours, respectively). Periodic IC_50_ determinations showed that the degree of auranofin resistance in the A2780/AF-R cell line was stable, during a 3-month period of subculture in drug-free medium (RI 20.7, 18.0 and 18.7 at 1, 2 and 3 months, respectively).

### Cross-resistance studies

**Figure 1:**
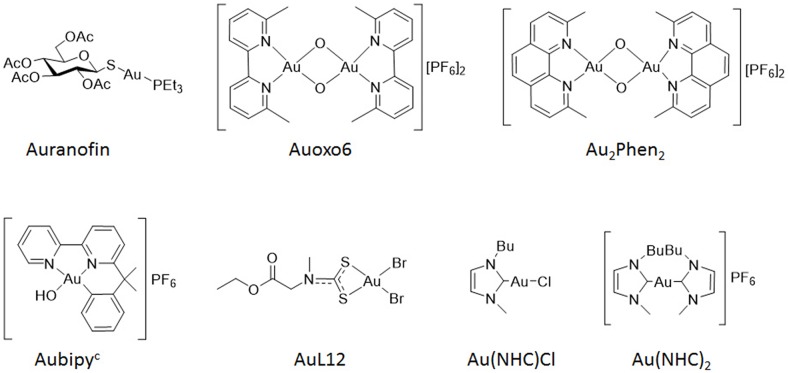
Chemical structures of gold (I) and gold (III) compounds

**Figure 2:**
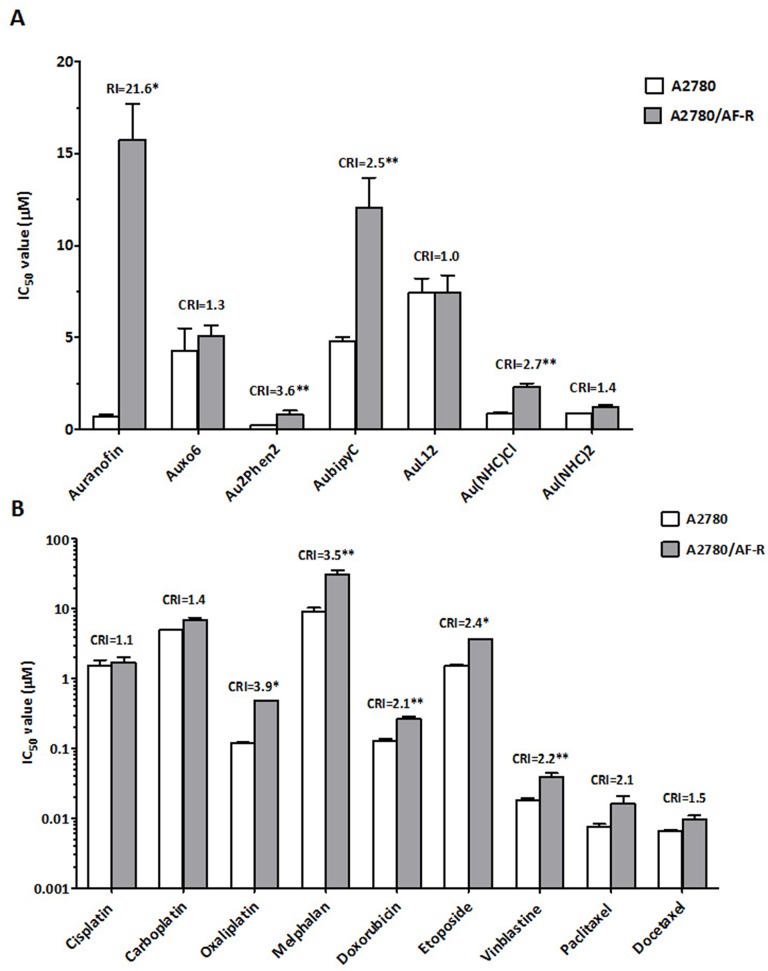
Comparative cytotoxicity of auranofin and other gold compounds (A) and classical chemotherapeutic agents (B) on cell growth of A2780 and A2780/AF-R cells Data are means ± standard error derived from at least three independent 72-h drug exposure experiments. CRI, cross-resistance index; ^*^p<0.001; ^**^p≤0.05.

An analysis of the pattern of sensitivity/resistance to other gold (I or III)–based compounds of more recent synthesis (Auoxo6, Au_2_Phen_2_, Aubipy^c^, AuL12, Au(NHC)Cl and Au(NHC)_2_) (Figure 1) was performed by concurrent comparisons of drug IC_50_ values of the auranofin resistant A2780 subline (A2780/AF-R) with those of the parental auranofin sensitive A2780 cell line. Only Au_2_Phen_2_ was more active, on a micromolar basis, than auranofin. In the sensitive cell line the relative degree of cytotoxicity was Au_2_Phen_2_>auranofin>Au(NHC)_2_>Au(NHC)Cl>Auoxo6>Aubipy^c^>AuL12 (most to least active) (Figure 2A).

The development of primary resistance to auranofin *in vitro* also resulted in a low level of cross-resistance to three of the tested gold compounds (Aubipy^c^, Au_2_Phen_2_, and Au(NHC)Cl, cross-resistance index (CRI) of 2.5, 3.6 and 2.7, respectively) but not to other three gold-compounds (Auoxo6, AuL12, and Au(NHC)_2_) after a 72-hour exposure. The latter compounds were able to completely circumvent resistance to auranofin (CRI <1.5) (Figure 2A).

Cross-resistance to other drugs commonly used in ovarian cancer therapy was assessed by concurrent comparisons of the IC_50_ values of the resistant subline with those of the parental line. Cross-resistance to these drugs variably accompanied development of *in vitro* primary resistance to auranofin in this line. The A2780/AF-R subline was modestly cross-resistant to oxaliplatin, melphalan, etoposide, vinblastine, doxorubicin and paclitaxel (CRI from 3.9 to 2.1, respectively). No cross-resistance to cisplatin, carboplatin and docetaxel was demonstrated (CRI≤1.5) (Figure 2B).

### Cellular gold accumulation

**Figure 3:**
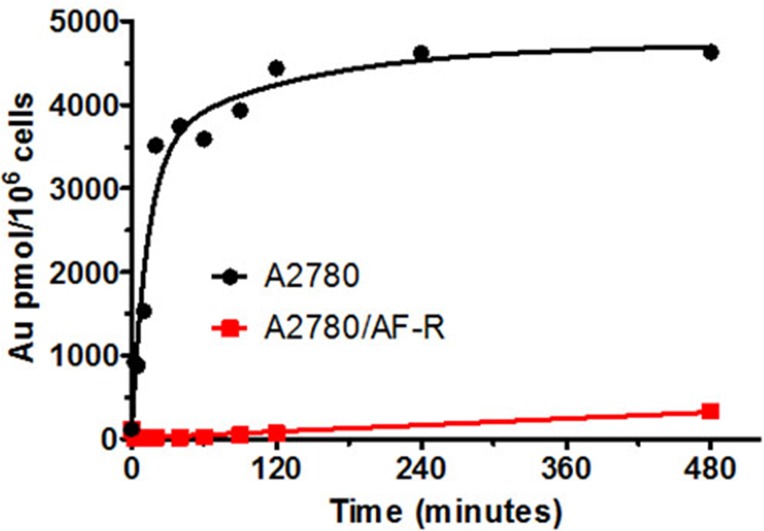
Cellular gold accumulation in A2780 and A2780/AF-R Cellular gold accumulation after exposure to 10μM auranofin ranging from 0 to 480 min in A2780 (black curve) and A2780/AF-R (red curve) cells. One representative experiment performed in duplicate.

To investigate whether resistance to auranofin in A2780/AF-R was related to a decrease in drug accumulation, cellular gold content was determined during an 8-hour exposure to 10 μM auranofin in both A2780/AF-R and A2780 (Figure 3). When auranofin cellular accumulation was examined, a substantial reduction in the content and in the AUC of gold was found in A2780/AF-R cells compared to that in A2780 sensitive cells (338.0 pmol/10^6^ cells *vs* 4630.0 pmol/10^6^ cells at 480 min, respectively, i.e. an 86.3% reduction; AUC_0-8hr_ 1.3×10^3^ *vs* 3.5×10^4^ pmol x hr/10^6^ cells, respectively, i.e. a 96.3% reduction). The accumulation of cellular gold was rapid and reached a plateau at about 120 min in A2780 cells. In contrast, the accumulation of cellular gold was dismal and displayed a linear kinetics during the entire drug exposure time in A2780/AF-R cells.

### mRNA expression levels of transport and trafficking pathway genes in A2780/AF-R and A2780

**Figure 4:**
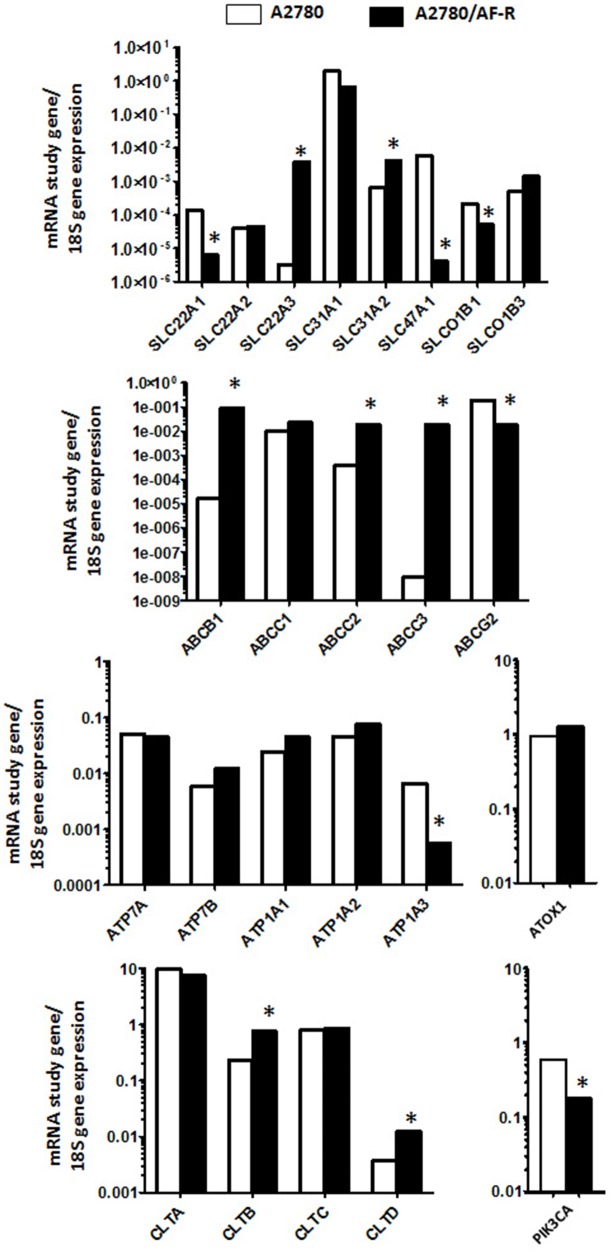
Up- and down-regulated genes in A2780/AF-R compared with A2780 cells Relative mRNA expression levels of study genes according to 18S mRNA in A2780 (white bars) and A2780/AF-R (black bars). ^*^>3-fold up or down-regulation of A2780/AF-R compared with A2780.

To verify whether changes in the expression of transport and trafficking pathway genes might be associated to the observed reduced gold accumulation in auranofin resistant cells, mRNA expression levels of candidate genes were measured in A2780/AF-R and A2780 cells. Overall, changes in mRNA expression of 13 out of 24 evaluated genes in A2780/AF-R cells compared to A2780 cells were observed. Seven were up-regulated (from 3.3 to > 2×10^6^-folds) and 6 down-regulated genes (from 3.4 to 1.4×10^3^-folds) (Figure 4, Table 1).

**Table 1:** Genes associated with resistance to auranofin as measured by RT-PCR analysis

Gene name	Description	Fold change
***Up-regulated genes***		
*ABCC3*	ATP binding cassette subfamily C member 3	2.1 x10^6^
*ABCB1*	ATP binding cassette subfamily B member 1	5.2 × 10^3^
*SLC22A3*	Solute carrier family 22 member 3	1.2 x10^3^
*ABCC2*	ATP binding cassette subfamily C member 2	48.0
*SLC31A2*	Solute carrier family 31 member 2	6.3
*CLTD*	Clathrin, heavy chain-like 1	3.4
*CLTB*	Clathrin, light chain B	3.3
***Down-regulated genes***		
*SLC47A1*	Solute carrier family 47 member 1	1.4×10^3^
*SLC22A1*	Solute carrier family 22 member 1	21.9
*ATP1A3*	ATPase, Na+/K+ transporting, alpha 3	11.6
*ABCG2*	ATP-binding cassette, sub-family G member 2	11.2
*SLCO1B1*	Solute carrier organic anion transporter family member 1B1	4.1
*PIK3CA*	Phosphatidylinositol-4,5-bisphosphate 3-kinase catalytic subunit alpha	3.4

Among genes belonging to the solute carrier family (SLC), a 21.9-fold decrease and a 1.2×10^3^-fold increase of mRNA levels of the organic cation transporter genes *SLC22A1* and *SLC22A3*, respectively, was observed in A2780/AF-R cells compared to the A2780 cells. Also, a 6.3-fold increase in the mRNA expression of the copper transporter protein *SLC31A2* was observed in A2780/AF-R cells compared to A2780 cells and a 1.4×10^3^-fold decrease of mRNA level of the multidrug/toxin extrusion transporter *SLC47A1* was observed in A2780/AF-R. No significant changes were observed in the other 3 solute carrier family genes analysed.

A substantial increase in mRNA expression levels of the ATP binding cassette transporters *ABCB1*, *ABCC2* and *ABCC3* (from 48 to >1×10^6^ folds) was observed in the resistant cell line compared with the sensitive one whereas a decrease of about 11-fold was observed in the mRNA of *ABCG2* in the auranofin resistant cell line. The highest increase was observed in *ABCC3*, as a consequence of its almost undetectable expression level in A2780 cells.

No substantial difference was observed between the expression of the copper transporters *ATP7A* and *ATP7B* or the Na^+^/K^+^ ATPase transporters *ATP1A1* and *ATP1A2* between the two cell lines with the exception of a 11.6-fold decrease in mRNA expression levels of *ATP1A3* in the resistant cell line compared with the sensitive one. No mRNA expression difference was observed for *ATOX1*.

Among *CLT* genes that mediate endocytosis, an about 3-fold increase in *CLTB* and *CLTD* gene expression was observed in the auranofin resistant cell line. An about 3-fold decrease was observed in *PIK3CA* expression in A2780/AF-R cells as compared to A2780 cells.

### Protein levels of selected relevant transporters

**Figure 5:**
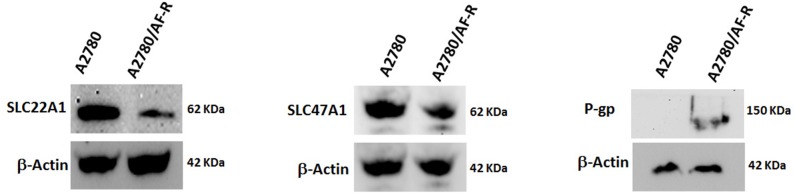
Variation of protein expression levels of selected transporters Protein levels of SLC22A1, SLC47A1 and P-gp were determined by Western blotting. β-actin was included as a loading control.

To determine whether the observed variations in mRNA levels of selected relevant transporters were correlated with variations at protein levels, Western blotting analysis of SLC22A1, SLC47A1 and P-gp was performed in A2780 and A2780/AF-R cells. Although less markedly, results confirmed mRNA observations. Downregulation of SLC22A1 and SLC47A1 proteins in A2780/AF-R compared with A2780 cells was observed. P-gp expression was negligible in A2780 sensitive cells but was detectable at low levels in A2780/AF-R cells (Figure 5).

### Reversal of auranofin resistance by verapamil

**Figure 6:**
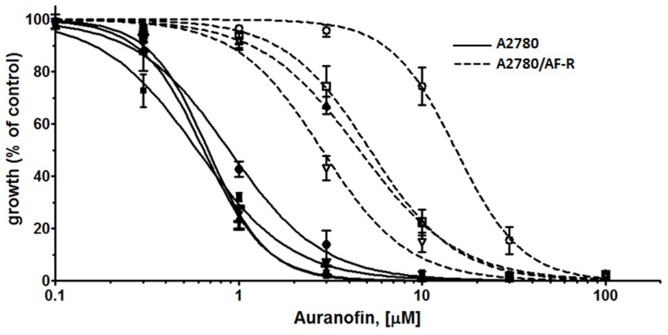
Modulation of P-glycoprotein-mediated resistance in A2780/AF-R and A2780 by verapamil Dose-response curves for auranofin (from 0.1 to 100 μM with or without verapamil (5, 10 or 20 μM) in A2780 (solid curves) and A2780/AF-R (dashed curves) cells are shown. Open or solid circles, 0 μM verapamil; open or solid squares, 5 μM verapamil; open or solid up triangles, 10 μM verapamil; open or solid down triangles, 20 μM verapamil. Data are means ± standard error derived from three independent 72-h drug incubation experiments.

Verapamil abrogates efflux of anticancer drugs mediated by P-glycoprotein (P-gp) by competitive inhibition of drug transport. Since the A2780/AF-R displayed characteristics of drug accumulation compatible with the classical multi-drug resistance phenotype mediated by the overexpression of P-gp and other ABC transporters, verapamil was used to investigate its ability to reverse auranofin resistance *in vitro*. Results showed that verapamil partially reverted the resistant phenotype. In A2780/AF-R, co-exposure of auranofin with verapamil 5, 10, 20 μM resulted in a 2.7-, 2.9- and 4.8-fold increase in the cytotoxicity of auranofin, respectively (Figure 6). No substantial differences in the growth inhibitory effects of auranofin were observed after co-exposure with verapamil in the auranofin sensitive cell line (i.e. 1.2-fold cytotoxicity increase following the addition of verapamil 5, 10, 20 μM) (Figure 6).

### Activity of thioredoxin reductase

To evaluate a potential role of thioredoxin reductase (TrxR) in the acquired resistance to auranofin, its activity was investigated in both cell lines. Results showed that cellular TrxR activity was moderately higher in A2780/AF-R cells compared with A2780 cells (1.8-fold variation) (Table 2).

**Table 2:** Basal thioredoxin reductase activity in A2780 and A2780/AF-R cells

Cell lines	TrxR activityunit/mg protein x min ± SD
**A2780**	2.751±0.665
***n***	*3*
**A2780/AF-R**	4.916±1.537
***n***	*3*

TrxR, thioredoxin reductase; SD, standard deviation; n, number of independent experiments

### Intracellular glutathione levels

To investigate whether putative increased intracellular glutathione levels may contribute to the development of resistance to auranofin, basal total and reduced glutathione was evaluated in the auranofin sensitive and resistant cell lines. No difference was observed in total and reduced intracellular glutathione levels in A2780/AF-R compared to A2780 cells (Table 3). These results do not show correlation between glutathione and auranofin resistance in A2780/AF-R cells characterized by a high transport deficit.

**Table 3:** Basal intracellular glutathione levels in A2780 and A2780/AF-R cells

Cell lines	GSH + GSSGμg/10^6^ cells ± SD	GSHμg/10^6^ cells ± SD
**A2780**	17.12±2,02	14.51±1.51
***n***	*3*	*3*
**A2780/AF-R**	17.76±2.08	15.39±1.53
***n***	*3*	*3*

GSH + GSSG, total glutathione; GSH, reduced glutathione; SD, standard deviation; n, number of independent experiments

## DISCUSSION AND CONCLUSIONS

The development of resistance to anticancer agents is the main determinant of failure of anticancer chemotherapy in initially drug sensitive cancers [29]. *In vitro* human cell lines resistant to anticancer drugs represent useful models to study the mechanisms of drug action and tumour resistance. This is the first investigation describing a human tumour cell line with acquired resistance to a gold compound.

The human ovarian A2780 cell line, intrinsically sensitive to cisplatin, was used to develop an auranofin resistant cell line. Following stepwise selection with auranofin, an about 22-fold resistant subline (A2780/AF-R) was obtained. Auranofin is currently undergoing clinical trial investigations in various types of cancers including ovarian cancer [10]. It also represents a reference compound when pharmacology of gold-based compounds is investigated.

The cytotoxic activity of a series of gold (I) and gold (III) compounds, platinum compounds and other cytotoxic anticancer agents characterized by different mechanisms of action against auranofin-resistant A2780/AF-R cells was tested. Resistance to auranofin in the A2780/AF-R cell line was associated with a low level of cross-resistance to three of the gold compounds tested (i.e. Aubipy^c^, Au_2_Phen_2_, and Au(NHC)Cl) as well as to oxaliplatin, melphalan, doxorubicin, etoposide, vinblastine, and paclitaxel. Lack of cross-resistance was observed against the other gold compounds and anticancer agents tested.

Knowledge of the cellular pharmacology of auranofin in experimental tumour models is still limited and little information is available on the mechanisms of tumour resistance to gold compounds [16]. The cytotoxic effects of auranofin appear to be directly related with the cellular content of auranofin-derived gold [5]. Also, in cultured human normal HE epithelial cells following stepwise selection for resistance to auranofin it was shown that maintenance of gold concentrations at a low level was a mechanism contributing to the observed cellular acquired resistance to auranofin [27]. Our data showed that intracellular gold accumulation during an 8-hour drug exposure time was markedly reduced in A2780/AF-R cells as compared to A2780 cells. This strongly suggests that the reduced sensitivity to auranofin is primarily due to a reduced steady-state cellular content of auranofin.

At present, only limited information is available on the mechanisms of intracellular uptake and accumulation of gold compounds [30]. Due to the ability of gold compounds to interact with thiol groups, transport through a thiol-shuttle has been suggested also involving reversible binding to serum albumin [31]. More recently, processes potentially involved in gold compound transport that include both influx and efflux systems have been hypothesized [32] on the basis of information available for platinum compounds [33, 34], Influx transporters comprising copper transporter (CTR) proteins and organic cation transporters (OCTs) are involved in the uptake of platinum compounds. Efflux transporters including adenosine triphosphate (ATP) binding cassette (ABC) multidrug transporters, copper-transporting P-type adenosine triphosphatases (ATPases) and multidrug and toxin extrusion (MATE) proteins are also involved in the extrusion of these metal compounds. Endocytotic processes mediated by clathrin vesicles or by Na^+^/K^+^ ATP-ases and micropinocytosis involving a Na^+^/H^+^ exchanger and PKI3CA in the signalling cascade have also been postulated to be involved in intracellular metal accumulation [32].

Our analysis of mRNA expression of genes potentially involved in cell transport or trafficking of gold compounds showed variations in 13 out of 24 genes in A2780/AF-R cells compared with A2780 cells. Seven genes were up-regulated and 6 genes were down-regulated.

It is known that the physiological role of members of the SLC family is heterogeneous since some of them are involved in the influx or efflux of organic cations or anions or in both these processes [35]. The organic cation transporters OCT1 and OCT2 (encoded by *SLC22A1* and *SLC22A2*, respectively) play an important role in the cellular uptake of some gold (I) N-heterocyclic carbene complexes (e.g. chlorido-[1,3-dimethyl-4,5-diarylimidazol-2-ylidene] gold complexes) [32].

We observed a downregulation of *SLC22A1* both at mRNA and protein expression level in A2780/AF-R cells compared to A2780 cells. These findings suggest that OCT1 (SLC22A1) may contribute to the uptake of auranofin and to resistance to this drug. In addition, it has been shown that oxaliplatin is an excellent substrate for OCT1 [36]. Thus, overexpression of *SLC22A1* could explain at least in part the observed cross-resistance to this drug in A2780/AF-R cells. While *SLC22A2* mRNA expression was unchanged, the expression of *SLC22A3* gene, encoding for OCT3, was higher in A2780/AF-R cells compared with A2780 cells. It has been suggested that oxaliplatin is transported by OCT3 [37] and that this transporter contributes to accumulation of this drug into cancer cells [38]. However, it has also been shown that intracellular accumulation of oxaliplatin is mainly due to increased expression of OCT2 rather than OCT3 or other OCTs as it occurs for other platinum compounds [39]. No information is available on the role of OCT3 in the transport of gold compounds or other anticancer drugs. Our results do not support a role of OCT3 in the resistance to auranofin.

The copper transporter CTR1, codified by *SLC31A1*, has also been suggested to be involved in the uptake and accumulation of gold (I) N-heterocyclic carbene complexes [32]. The expression of *SLC31A1* was found slightly downregulated (2.9-fold) in A2780/AF-R cells compared to A2780 cells. This could be related to the reduced gold accumulation and cytotoxicity of auranofin observed in A2780/AF-R cells. The role of CTR2 in the uptake and accumulation of gold compounds is currently unknown. A modest upregulation of *SLC31A2* gene expression in A2780/AF-R cells compared to A2780 cells was observed. The contribution of this gene up-regulation to the accumulation and cytotoxicity of auranofin remains undefined.

Cisplatin and carboplatin [33, 40] and to a lesser extent oxaliplatin [41] are substrate of CTR1. Cisplatin and carboplatin are also substrates of CTR2. No clear correlation between observed CTR1 and CTR2 expression levels and sensitivity/resistance patterns to platinum compounds tested can be established.

MATE transporters are responsible for the excretion of xenobiotic organic cations in the kidney and liver. MATE1 encoded by *SLC47A1* is also expressed in other tissues and is involved in the renal accumulation and nephrotoxicity of platinum agents [42]. The expression of *SLC47A1* gene and that of its transcript was down-regulated in A2780/AF-R cells compared with A2780 cells. This data may explain the reduced cytotoxicity of oxaliplatin in A2780/AF-R cells and suggests a potential role of MATE1 also in auranofin transport and resistance.

Organic anion transporting polypeptides (OATPs) belonging to the SLCO subfamily play a relevant role in transporting endo- and xenobiotics, including several anticancer drugs, across plasma membranes. *SLCO1B3* gene overexpression was associated with increased cellular accumulation of platinum and increased sensitivity to platinum anticancer drugs in human tumour cell lines [43]. No difference in the expression of *SLCO1B3* in A2780/AF-R cells compared with A2780 cells was observed. A decrease in the expression of *SLCO1B1* in A2780/AF-R cells compared with A2780 cells was instead shown. Although the role of SLCO1B1 in the transport of metal compounds has not been investigated, a contribution of downregulation of this carrier to tumour resistance to auranofin and oxaliplatin in A2780 cells cannot be ruled out.

ATP binding cassette transporters are members of a large family of cell membrane efflux transporters. Overexpression of these transporters confers resistance to a large series of structurally unrelated anticancer drugs [44].

P-gp, codified by the *ABCB1* gene, is responsible for the development of the multidrug resistance cancer phenotype [45, 46]. Increased *ABCB1* mRNA expression level in A2780/AF-R cells as compared to A2780 cells was observed. This was confirmed also at the protein level. Auranofin sensitivity was partially restored after co-exposure of the resistant cell line to verapamil and auranofin. These findings suggest a contribution of this transporter in the efflux of auranofin and in the development of acquired resistance to this drug. Little information is available on the role of P-gp in the cellular kinetics and activity of gold compounds. A recent published study showed that an investigational carbene gold compound was active in two human leukemia sublines resistant to either vincristine or daunorubicin, due to overexpression of P-gp [47]. No difference in the gold uptake between these resistant leukaemia cell lines and their sensitive counterpart was observed, conveying that the study gold compound was not a P-gp substrate [47]. These data suggest that different gold compounds may undergo different mechanisms of drug efflux.

Since *ABCB1* overexpression is involved in tumour resistance to several anticancer drugs including vinca alkaloids, doxorubicin, etoposide and taxanes, low grade cross-resistance to some of these drugs observed in A2780/AF-R cells might be mediated by increased P-gp levels.

Similar considerations may be done for cross-resistance to oxaliplatin and melphalan observed in A2780/AF-R cells. In colon cancer cell lines the overexpression of *ABCB1* was associated to oxaliplatin resistance [48]. Also, increased *ABCB1* expression and decreased intracellular melphalan accumulation as well as increased resistance to this drug in a human HL-60 promyelocytic leukaemia cell line have been reported [49].

We observed an increase in the mRNA expression of *ABCC2*, a member of the MRP subfamily, in A2780/AF-R cells as compared to A2780 cells. In tumour models the overexpression of this transporter has been associated with the development of tumour resistance to several anticancer agents including platinum compounds, vinblastine and etoposide [50]. In keeping with these data, resistance to these drugs was observed in A2780/AF-R cells. A high level of *ABCC3* expression was observed in A2780/AF-R cells as compared to A2780 due to the negligible expression of this gene in A2780 cells. Resistance to etoposide has been associated to *ABCC3* overexpression [51], thus resistance to this drug in A2780/AF-R cells may be in part due to this occurrence.

Our association data suggest a role of these ATP binding cassette transporters also in the development of acquired resistance to auranofin.

Breast cancer resistance protein (BCRP) encoded by *ABCG2* displays an important role in resistance to several anticancer drugs, including camptothecins, antifolates and anthracyclines. We observed down-regulation of *ABCG2* in A2780/AF-R cells compared to A2780 cells but no meaningful association could be established with observed sensitivity/resistance patterns. The involvement of BCRP in the transport of platinum and other metal drugs has in fact been not ascertained. In particular, contradictory results have been reported on the role of this transporter in the development of oxaliplatin resistance.

Following uptake by CTRs, intracellular copper and other metals are tightly controlled and shuttled through subcellular compartments via interactions with copper chaperones and secreted. Homeostasis of copper is maintained by antioxidant 1 (ATOX1) that transfers copper to the copper transporting P1B-type ATPases ATP7A and ATP7B. It has been shown that overexpression of ATOX1, ATP7A and ATP7B reduces cisplatin accumulation and sensitivity [52–54]. No substantial differences in the mRNA expression of these genes were observed in A2780/AF-R cells compared to the A2780 cells and no association with resistance to auranofin was ascertained.

*ATP1A1,* *ATP1A2* and *ATP1A3* genes encode for isoforms of Na^+^/K^+^-ATPase, a membrane protein responsible the electrochemical Na^+^/K^+^ gradients essential for Na^+^-coupled transport of various organic and inorganic molecules. We observed downregulation of *ATP1A3* gene expression and no significant change in the mRNA expression of *ATP1A*1 and *ATP1A2* genes in A2780/AF-R cells compared to A2780 cells. Very limited data are available on the involvement of these proteins in the transport of anticancer agents including metal drugs. However, it has been shown that inhibition of Na^+^/K^+^-ATPase with ouabain reduces platinum accumulation in A2780 cells and that lower expression of Na^+^/K^+^-ATPase alpha-1 subunit is associated with resistance to cisplatin [55]. Our results do not allow to speculate on the potential role of these proteins in the resistance to auranofin and other drugs tested.

To verify the hypothesis that selective inhibition of clathrin function reduces cytotoxic activity of gold compounds as a consequence of reduced gold uptake through clathrin-mediated endocytosis [32], we investigated the potential association of the expression of clathrins A (*CLTA*), B (*CLTB*), C (*CLTC*) and D (*CLTD*) with auranofin resistance. The observed modest increase of *CLTB* and *CLTD* expression levels in the auranofin resistant cell line compared with the auranofin sensitive cell line do not support the hypothesis that gold uptake may be mediated by clathrin endocytosis.

It was also shown that a modest attenuation of the cytotoxicity of some gold carbene complexes occurred upon addition of wortmannin, a fungal metabolite inhibitor of PIK3CA [32]. Since activated PIK3CA is involved in the signalling cascade leading to macropinosome formation, it was speculated that this finding may be due to specific inhibition of this putative mechanism of gold compound uptake. However, no information is available on its role in the transport of anticancer agents with the exception of macromolecular conjugated drugs. Also, PIK3CA is not only involved in intracellular trafficking but also in several other processes including cell growth, proliferation, differentiation, motility and survival. The mRNA expression of *PIK3CA* was modestly decreased in A2780/AF-R cells compared to A2780 cells. This borderline association does not allow us to conclude that macropinocytosis play a role in the uptake of gold compounds.

Due to the recognized ability of auranofin to inhibit TrxR, we verified if alterations in TrxR enzymatic activity may contribute to the mechanism of acquired resistance to auranofin. The moderately higher activity (< 2-fold) observed in the A2780/AF-R cells compared with A2780 cells might reflect a concurrent adaptative cellular response to stepwise selection with increasing auranofin concentrations during time, while high level resistance developed due to transport defects. *In vitro* tumour sensitivity and resistance to auranofin and platinum compounds have been associated to changes in intracellular TrxR activity. High level (≥ 10-fold) intrinsic resistance to auranofin has been associated to a substantial increase in TrxR activity in non-small cell lung cancer cell lines [56]. Also, low-intermediate level acquired resistance (about 2-5-fold) to cisplatin associated to slight-moderate increases (1.3-2-fold) in TrxR activity have been reported in various tumour cell lines [57–59]. No cross-resistance with cisplatin was however observed in our model. It is conceivable that this mechanism may have contributed at least in part to auranofin resistance observed in A2780/AF-R cells.

Thiols may play an important role in the molecular mechanisms of resistance to anticancer drugs, including metal drugs. Glutathione is the main cellular thiol that interacts with copper and platinum-based drugs. It is common to find elevated glutathione levels in cisplatin resistant tumour models [60]. No differences in intracellular glutathione content between A2780/AF-R cells and A2780 cells were observed. Thus, no contribution of this mechanism of resistance was noted.

Our results support the knowledge that transport of auranofin is a critical determinant of cytotoxicity of this drug in cancer cells. An about complete abolition (>95%) of gold accumulation occurred in A2780/AF-R cells. This was associated to a high level (>20 fold) of acquired resistance. The only previously reported data do not relate to a tumour model (HE cells) [27]. In this study, the HE auranofin-resistant subline was selected by a stepwise process similar to that used in the present investigation, reaching a somewhat lower drug concentration (2 μM). Following a 24-h auranofin exposure, cellular gold content observed in auranofin-resistant HE cells were only one-half of those observed in parental HE cells.

The observed highly reduced accumulation of gold in A2780/AF-R cells was associated to expression changes of various influx and efflux transporters. In addition, verapamil reverted only partially resistance to auranofin at concentrations able to abrogate almost completely vinblastine and doxorubicin resistance in the classical multidrug resistance phenotype. This provides evidence that multiple drug transporters may act as mediators of auranofin and possibly of other gold compound transport in cancer cells. However, the relative contribution of the single transporters investigated in this study remains to be settled by future studies. An additional contribution to the degree of auranofin resistance by the observed moderately increased TrxR activity may not be ruled out.

Our findings warrant further investigation into the molecular mechanisms mediating transport of auranofin in view of a potential role of this agent in the treatment of human cancer. The understanding of gold transportome by means of transcriptomics analysis is ongoing at our laboratory as well as the attempt to evaluate the relative contribution of single processes potentially involved by functional studies with specific transport inhibitors or gene silencing of transporters and protein expression studies. This will provide crucial insights into the cellular pharmacology of auranofin and information useful for the design of new gold complexes for clinical application.

## MATERIALS AND METHODS

### Drugs and supplies

Auranofin was obtained from Vinci Biochem (Vinci, Italy); Auoxo6 [61] and Au_2_Phen_2_ [62], two dinuclear gold (III) compounds, and Aubipy^c^ [63], an organogold (III) complex, were provided by Prof. M.A. Cinellu. AuL12 [64], a gold (III) dithiocarbamate complex, was provided by Prof. D. Fregona. The two gold (I) carbene compounds, namely chlorido (1-butyl-3-methyl-imidazole-2-ylidene) gold (I) (Au(NHC)Cl)) and bis(1-butyl-3-methyl-imidazole-2-ylidene) gold (I) (Au(NHC)_2_) [65], were provided by Prof. C. Gabbiani. Cisplatin, oxaliplatin, carboplatin, melphalan, vinblastine, etoposide, doxorubicin, paclitaxel, docetaxel, verapamil and sulforhodamine B (SRB) were obtained from Sigma (Milan, Italy). All the other chemicals were of analytical grade. RPMI 1640 cell culture medium, foetal calf serum (FCS), and phosphate-buffered saline were obtained from Celbio (Milan, Italy).

### Cell lines and culture conditions

The human auranofin-sensitive parental ovarian cancer A2780 cell line was a gift of Prof. Kevin Scanlon (Keck Graduate Institute, CA, U.S.A). The auranofin-resistant cell subline (A2780/AF-R) was established from the parental sensitive cell line as described in the following paragraph. Cell lines were maintained in RPMI-1640 supplemented with 10% FCS and antibiotics (penicillin, 100 U/ml; streptomycin, 100 μg/ml) at 37°C in a 5% CO_2_ humidified atmosphere and subcultured twice weekly.

### Development of resistance to auranofin

A2780/AF-R cells were obtained by a continuous exposure of the parental sensitive A2780 cells to stepwise increasing auranofin concentrations. Initially, parental sensitive cells were grown in standard medium containing a low (sublethal) concentration of auranofin (100 nM). After 3 or 4 passages in this growth condition, auranofin concentration was increased in a stepwise manner up to 6 times until a 7 μM final concentration. This concentration level was achieved in an 8-month selection period.

### Cytotoxicity and drug resistance reversal assays

The growth inhibitory effects of auranofin as well as of other six gold compounds and selected cytotoxic drugs against A2780/AF-R cells were determined by the SRB assay [66] to assess the degree of acquired resistance to auranofin and the potential occurrence of cross-resistance or collateral sensitivity to other drugs. Also, the potential modulation of resistance to auranofin by verapamil, a calcium channel blocker capable of reversing the classical multidrug resistance phenotype, was determined by the same assay.

Exponentially growing cells were seeded in 96-well plates in RPMI 1640 supplemented with 10% FCS at a plating density of 4×10^3^ cells/well.

After 24 hours, A2780 and A2780/AF-R cells were exposed to increasing concentrations of gold compounds or other drugs (cisplatin, carboplatin, oxaliplatin, melphalan, vinblastine, doxorubicin, paclitaxel, docetaxel and etoposide). Concentrations ranged from 1 nM to 100 μM and each concentration was tested in triplicate.

To assess the reversal of resistance to auranofin by verapamil, cells were pre-treated with this drug 15 min before auranofin exposure and co-exposed to verapamil during auranofin exposure. Concentrations of verapamil used (i.e. 5, 10, 20 μM) were not cytotoxic (lower than IC_20_).

In all cases, after 72-hour drug exposure, cells were fixed with 10% trichloroacetic acid and stained with 0.4% SRB in 1% acetic acid. The SRB fixed to the cells was dissolved in 10 mM Tris-HCl and absorbance was read on an automated plate reader at a wavelength of 540 nm.

The IC_50_ drug concentration resulting in a 50% reduction in the net protein content (as measured by SRB staining) in drug treated cells as compared to untreated control cells was determined. All the reported IC_50_ values represent the mean of at least three independent experiments.

The degree of acquired resistance to auranofin in A2780/AF-R cells was calculated as the ratio between IC_50_ values of A2780/AF-R and the IC_50_ value of the parental cell line (resistance index, RI).

The degree of cross-resistance to the other gold compounds or cytotoxic agents tested in A2780/AF-R cells was calculated as the ratio between IC_50_ values of A2780/AF-R and those of the parental cell line (cross-resistance index, CRI).

In the experiments of auranofin resistance reversal, the reversal fold (RF) values indicating the potency of reversal were calculated by dividing IC_50_ of auranofin alone by IC_50_ of auranofin in combination with verapamil.

### Determination of cellular gold accumulation

A2780/AF-R and parental A2780 cells were seeded in duplicate in 60 mm^2^ Petri dishes in RPMI 1640 supplemented with 10% FCS at plating densities of 2×10^6^ cells/dish following a 7-day culture period in drug-free medium.

After 24 hours, auranofin was added at high concentration (10 μM), then cells were incubated for 0, 2.5, 5, 10, 20, 40, 60, 80, 120, 240, and 480 min. The cell monolayers were washed 3 times with PBS and, after trypsinization, cells were pelleted, counted and then lysed by cell lysis buffer.

The gold content was determined following a previously published protocol [67] with some slight modifications. Each sample of pellet was recovered in a PE vial and digested in a thermo-reactor at 80°C for 8 h with 1.0 mL of HNO_3_ 69.5% suprapure grade. After digestion, samples were diluted exactly to 6 mL with ultrapure water (≤18 MΩ). The determination of gold content in these solutions was performed in duplicate by a Varian 720-ES Inductively Coupled Plasma Atomic Emission Spectrometer (ICP-AES). The calibration curve of gold was obtained using known concentrations of a gold ICP standard solution purchased by Sigma-Aldrich. Moreover, each sample were spiked with 1 ppm of Ge used as an internal standard. The wavelengths used for Au determination were 242.795 and 267.595 nm whereas for Ge the line at 209.426 nm was used. The operating conditions were optimized to obtain maximum signal intensity and, between each sample, a rinse solution containing 1.0 mL of HNO_3_ 69.5% suprapure grade and 5.0 mL of ultrapure water was used in order to avoid any “memory effect”.

The cellular gold content was calculated as the mean of two independent determinations and referred as total pmol Au/10^6^ cells. The area under the curve (AUC) of intracellular gold content was calculated by Graph Pad Prism Software (Inc., La Jolla, CA).

### Gene expression analysis

Overall, we analysed the expression of 24 mRNA genes involved in cellular transport and/or trafficking pathways. Genes were candidate on the basis of their known role in cell influx and/or efflux of metals (e.g. copper), metal-based anticancer drugs (e.g. platinum drugs), and more in general in tumour drug resistance. According to such criteria we evaluated the gene expression of 8 transporters belonging to the solute carrier family (*SLC22A1, SLC22A2, SLC22A3, SLC31A1, SLC31A2, SLCO1B1, SLCO1B3* and *SLC47A1*), 5 ATP-binding cassette transporters (*ABCB1, ABCC1, ABCC2, ABCC3, ABCG2*), 2 copper transporter ATPases (*ATP7A, ATP7B*), one copper chaperone (*ATOX1*), and 3 isoforms of Na^+^/K^+^ ATPase (*ATP1A1, ATP1A2, ATP1A3*). In addition, we analysed the mRNA expression of 4 clathrin isoforms (*CLTA, CLTB, CLTC, CLTD*), that are physiologically involved in mechanisms of internalization (e.g. endocytosis), and of *PI3KCA* gene that plays a key role in the signalling cascade associated with macropinocytosis. To identify genes with biologically significant expression changes, a 3-fold change was considered as an arbitrary cut-off [68].

### RNA preparation and amplification

Total RNA was isolated from A2780 and A2780/AF-R cell lines as previously described using a NucleoSpin^®^ RNA Protein Kit (Macherey-Nagel, Germany) according to instruction's manual. The RNA was quantified by Qubit™ 3.0 Fluorometer (Invitrogen, USA). 500ng of total RNA were retro-transcribed using iScript (Bio-Rad, USA) and amplified with specific primers listed in Table 4. All primers were purchased from IDT (IDT, Germany). PCR amplification was carried out by means SsoAdvanced™ Universal SYBR^®^ Green Supermix (Bio-Rad, USA) according to manual instruction using the RotorGene 3000 Instrument (Qiagen Germany). Ribosomal 18s rRNA was used as the normalizer. Quantitative PCR was performed using the following procedure: 98°C for 2 min, 40 cycles of 98°C for 5 sec, 60°C for 10 sec. The program was set to reveal the melting curve of each amplicon from 60°C to 95°C with a read every 0.5°C.

**Table 4:** Primers of study genes and of 18S normalizer gene used in RT-PCR

Genes	Forward	Reverse
*ABCB1*	CAGCTATTCGAAGAGTGGGCACAAAC	GCCTCTGCATCAGCTGGACTGTTG
*ABCC1*	ACGCTCAGAGGTTCATGGACTTGGC	CTCTTCATGTGGGCCACCTGATACG
*ABCC2*	GATCTCCTTTGCAAGTGACCGTGACAC	GGCCAAGTTGGATAGGGTCAATGCC
*ABCC3*	CTTGGCCTGCTTCAGGGAGAAACCT	CCACCATCTGGGATCTGTCCTCTTC
*ABCG2*	GACTCCAAGGTTGGAACTCAGTTTATCC	ATGGAGAAGATGATTGTTCGTCCCTGC
*ATOX1*	GACCTGCCCAACAAGAAGGTCTGCA	TGACTGCCAAGTCCCAGGTCTGTC
*ATP1A1*	GGCTGCAAGGTGGATAACTCCTCG	CCCAGAAGCAAGTGTGGCAATTCTTCC
*ATP1A2*	AACGAGACTGTGGAGGACATTGCAGC	CCCTCCACAATGATGAGCTTCTGCTG
*ATP1A3*	CTGTCATCTTCCTCATCGGCATCATCG	CTGTCATGCGGTTCTGAGTGAGGG
*ATP7A*	CCATTGCCACCCTCTTGGTATGGATTGT	CTGTACCCACCATCACAGCAGTTGG
*ATP7B*	CCACAGTGAGCGCCCTTTGAGTGC	TGGCTGTCTGTCCTTTCATCTCGTGG
*CLTA*	CTGAAGAGCCACCCTGTGGAAACAC	TCCTCCTCTCCCTCCTCTCTGAATG
*CLTB*	GATGTGTTTCAGGAGGCCAACGGTC	TGGTTCCACTCCTCCAGGTCCTTC
*CLTC*	TAACAACCCGGAGAGATTTCTTCGTG	TCCTTTCGACGTACCAGGTAGCGAG
*CLTD*	GTGTGTGCAGGTGGCCTCTAAGTAC	ATCCTCTCCACCTCCTTGATCTGCC
*PIK3CA*	GTCTGTCAATCGGTGACTGTGTGGG	CCAGCACATGAACGTGTAAACAGGTCAA
*SLC22A1*	CAACCTGGTGTGTGCTGACTCCTG	AAGAGCAGCATGGACATGTAGTTGGGC
*SLC22A2*	TGTTGGGCGGAGATATCGGAGAACAG	TATTCTGGGAGATCAGCCACCTGGG
*SLC22A3*	GTGTCAATGCGTGGATGCTGGACC	GCAGGAAGCGGAAGATCACAAACACA
*SLC31A1*	CTGAGCTTTCCTCACCTCCTGCAAAC	GCCATAGAGTTTGATGTCAATGGCAATGC
*SLC31A2*	AAGGCATCAAGGTTGGCAAAGCCAAGC	CCGATGACCACCTGGATGACATGG
*SLC47A1*	GTCACGATCTTCATTCCAGCTCTTCC	ACTGGGAAATCAAGTTTGCCAGTGCAG
*SLCO1B1*	TTTCTCTGCACTTGGAGGCACCTCAC	CATGACCCACGTGTGCCACAGTTGT
*SLCO1B3*	CCCTCTAATCTGCGAAAGCAAATCAGTTGCCGG	GAGGATTTGCATCCTGCTAGACAAGGTGAC
*18S*	CGGCTACCACATCCAAGGAA	GCTGGAATTACCGCGGCT

### Western blot analysis

60 μg of total proteins were separated by NuPAGE 12% Bis-Tris Gel and electroblotted (Turbo-Blot apparatus; Bio-Rad, USA) onto nitrocellulose membranes (Bio-Rad, USA) according to instruction's manual. The following antibodies were used: SLC22A1, SLC47A1, ABCB1 and β-Actin (GeneTex, USA). The membranes were incubated over-night at 4C°. After incubation membranes were washed with TPBS and incubated 1 h in TPBS/5% milk containing the corresponding peroxidase-conjugated secondary antibody (1:2000) (Bio-Rad, USA). After washing in TPBS, ECL (Bio-Rad, USA) was used to visualize the peroxidase-coated bands by means of ChemiDoc MP Imaging System (Bio-Rad, USA). Densitometric analysis was performed by Quantity One software (Bio-Rad, USA).

### Glutathione assay

Glutathione content in A2780/AF-R and A2780 cells was determined by a spectrophotometric method. Briefly, cells were seeded in 60 mm^2^ Petri dishes at a plating density of 1×10^6^ cells/well and incubated for 48 h. Cells were harvested and counted, the levels of intracellular total glutathione (GSH + GSSG) and of the reduced glutathione form (GSH) were measured using ApoGSH™ Glutathione Colorimetric Detection Kit (BioVision, California, USA).

Serial dilutions of GSH + GSSG and GSH were used to generate corresponding standard curves. GSH + GSSG and GSH concentrations were expressed as μg/10^6^ cells.

### Thioredoxin reductase activity

Thioredoxin reductase activity was determined spectrophotometrically in cell extracts by the Thioredoxin Reductase Assay Kit (Sigma-Aldrich, St. Louis, MO). A2780 and A2780/AF-R cells were seeded in 100 mm^2^ Petri dishes at a plating density of 2×10^6^ cells/well and incubated for 48 h. Cells were harvested, counted and washed with PBS. Samples were lysed with a 500 μl CelLytic Extraction Reagent (Sigma-Aldrich, St. Louis, MO) containing 50 μl of a protease inhibitor cocktail (Sigma-Aldrich, St. Louis, MO). After 15 min of incubation on a shaker, lysates were centrifuged at 12,000 g for 15 min to pellet cell debris and the obtained supernatants were tested for enzyme activities. Enzymatic activity was assessed by measuring the absorbance at 412 nm using a microplate reader. The Bradford Reagent (Sigma-Aldrich, St. Louis, MO) was used to determine protein concentrations. Results are the mean of three independent experiments.

### Statistical analysis

t-Tests were used for pairwise comparisons. P values <0.05 were considered statistically significant. All data were analysed using Graph Pad Prism version 5 (Graph Pad Prism Software Inc., La Jolla, CA).
